# Rapid and reliable detection of α-globin copy number variations by quantitative real-time PCR

**DOI:** 10.1186/2052-1839-14-4

**Published:** 2014-01-24

**Authors:** Runa M Grimholt, Petter Urdal, Olav Klingenberg, Armin P Piehler

**Affiliations:** 1 Department of Medical Biochemistry, Oslo University Hospital, Ullevaal, 0424 Oslo, Norway; 2 Department of Medical Biochemistry, Oslo University Hospital, Rikshospitalet, 0424 Oslo, Norway; 3 Fürst Medical Laboratory, 1051 Oslo, Norway

**Keywords:** α-thalassemia, Copy number variation, Quantitative real-time PCR, α-globin gene cluster, HS-40, Unknown deletions

## Abstract

**Background:**

Alpha-thalassemia is the most common human genetic disease worldwide. Copy number variations in the form of deletions of α-globin genes lead to α-thalassemia while duplications of α-globin genes can cause a severe phenotype in β-thalassemia carriers due to accentuation of globin chain imbalance. It is important to have simple and reliable methods to identify unknown or rare deletions and duplications in cases in which thalassemia is suspected but cannot be confirmed by multiplex gap-PCR. Here we describe a copy number variation assay to detect deletions and duplications in the α-globin gene cluster (HBA-CNV).

**Results:**

Quantitative real-time PCR was performed using four TaqMan® assays which specifically amplify target sequences representing both the α-globin genes, the –α^3.7^ deletion and the HS-40 region. The copy number for each target was determined by the 2^-ΔΔCq^ method. To validate our method, we compared the HBA-CNV method with traditional gap-PCR in 108 samples from patients referred to our laboratory for hemoglobinopathy evaluation. To determine the robustness of the four assays, we analyzed samples with and without deletions diluted to obtain different DNA concentrations. The HBA-CNV method identified the correct copy numbers in all 108 samples. All four assays showed the correct copy number within a wide range of DNA concentrations (3.2-100 ng/μL), showing that it is a robust and reliable method. By using the method in routine diagnostics of hemoglobinopathies we have also identified several deletions and duplications that are not detected with conventional gap-PCR.

**Conclusions:**

HBA-CNV is able to detect all known large deletions and duplications affecting the α-globin genes, providing a flexible and simple workflow with rapid and reliable results.

## Background

Copy number variations (CNVs) in the human genome are important genetic variations where large segments of DNA (> 1 Kb) are present in a variable number of copies in comparison to a reference genome [1]. A variety of processes can result in CNVs, including deletions, duplications and translocation during meiosis [1]. CNVs may influence both mRNA and protein levels and have recently been associated with several complex and common diseases [1,2].

Thalassemia is one of the most common human genetic diseases worldwide and is caused by reduced or absent production of globin chains, mainly of the α- or β- globin chains resulting in α- or β-thalassemia, respectively [3]. Most commonly, α-thalassemia is the result of the deletion of one or both of the α-globin genes, HBA1 and HBA2, located in the telomeric region on chromosome 16 (16p13.3). Point mutations that inactivate one of the linked α-globin genes are less frequent, but these non-deletional α-thalassemia variants may give rise to more severe reduction in α-chain production than deletion of one α-globin gene [4]. Reciprocal recombination during meiosis have caused the common -α^3.7^ and –α^4.2^ deletions and the α-globin gene triplications, ααα^anti3.7^ and ααα^anti4.2^[5]. The α-globin gene triplications most often have no phenotypic effect, but co-inheritance of an α-globin gene triplication and β-thalassemia trait may lead to more pronounced imbalance between α- and β-globin chains and β-thalassemia intermedia [6]. Duplication of the complete α-globin gene cluster has also been identified, increasing the number of α-globin genes from 4 to 6 in heterozygotes [7,8]. The expression of the α-globin genes are mainly regulated by the erythroid-specific DNase I hypersensitive site HS-40, located 40 Kb upstream of the sequence encoding the ζ-globin mRNA cap site [9]. Deletions involving only the HS-40 region give rise to a particular category of α-thalassemia with intact but functionally inactive α-globin genes [9].

Seven of the most common deletions that cause α-thalassemia (-α^3.7^, -α^4.2^, –^SEA^, –^FIL^ –^MED^, -(α)^20.5^ and –^THAI^) can easily be detected by multiplex gap-PCR [10,11]. However, there are several α-thalassemia associated deletions that will not be identified with this method [12]. Nor will co-inheritance of α-globin gene triplications in beta-thalassemia carriers be identified.

Several methods have been developed to identify unknown deletions and duplications in the α-globin genes, using quantitative real-time PCR (real-time qPCR) assays [13-15] or multiplex ligation-dependent probe amplification (MLPA) assays [16,17]. Here, we describe a fast, robust and specific method, designated HBA-CNV, that measures copy number variation using real-time qPCR. The method is designed to detect all larger deletions and duplications affecting the two α-globin genes and the regulatory region, HS-40.

## Results and discussion

**Figure 1:**
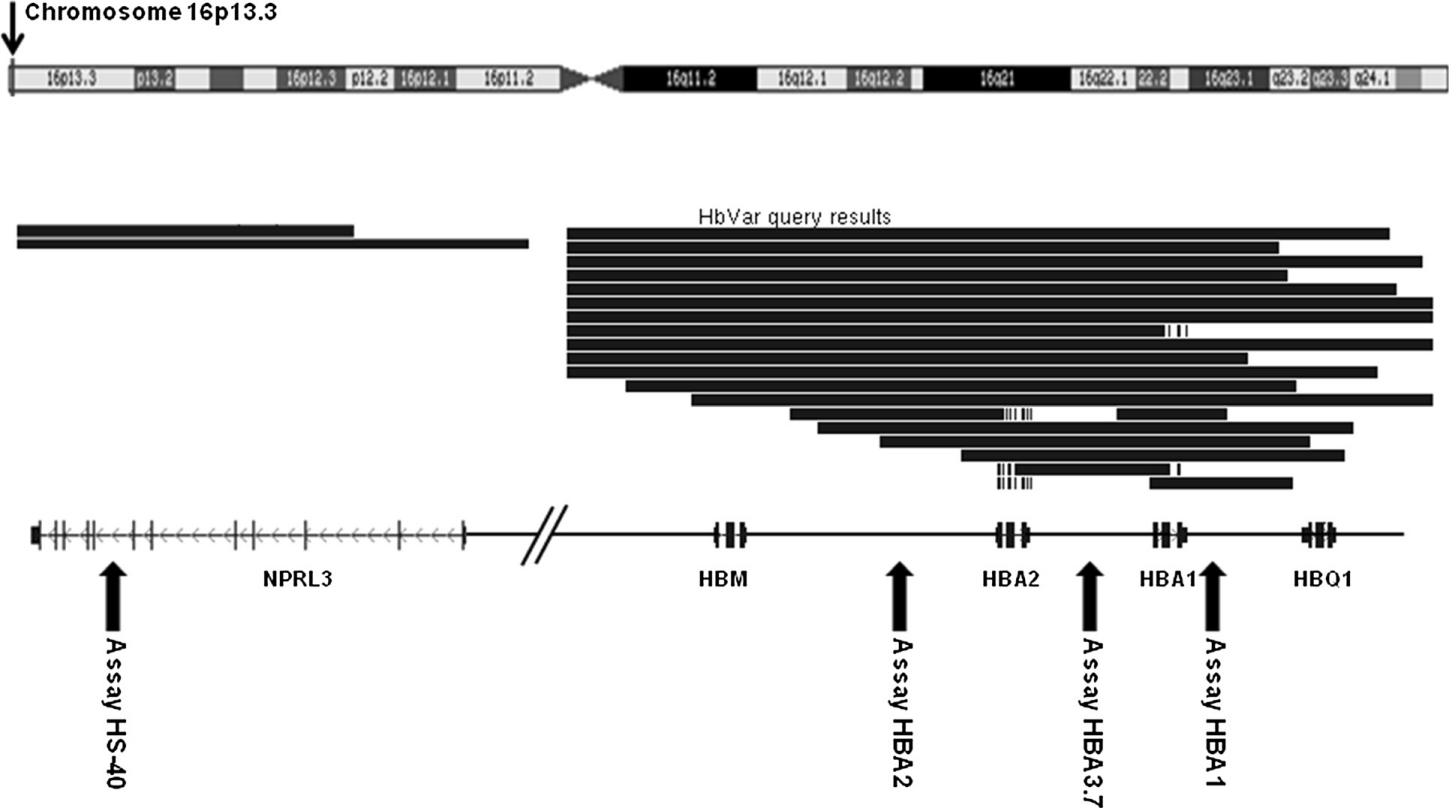
**Location of the four CNV assays.** Primers and probes were designed to efficiently amplify specific non-homologous regions in the α-globin gene cluster at the telomeric end of chromosome 16 (16p13.3). An assay located in intron 7 of the NRPL3 gene detects CNVs in the HS-40 element, which plays an important role in regulation of the α-globin genes. Black solid lines indicate known large deletions in the α-globin gene cluster, whereas small, blue dots indicate single nucleotide substitutions and small insertions and deletions that give rise to either hemoglobin variants or α-thalassemia.

Using the genome browser software with the implemented HbVar track, four positions were chosen for amplification (Figure 1). A pre-design TaqMan® Copy Numbers Assay was chosen to detect deletions affecting HBA1. In addition, three primer pairs and probes were designed to specifically amplify target sequences representing the HBA2 gene, the –α^3.7^ deletion and the HS-40 region. In addition to novel, larger deletions of the α-globin cluster, this method was designed to detect all known large deletions affecting one or both α-globin genes and their regulatory region causing α-thalassemia.

**Figure 2:**
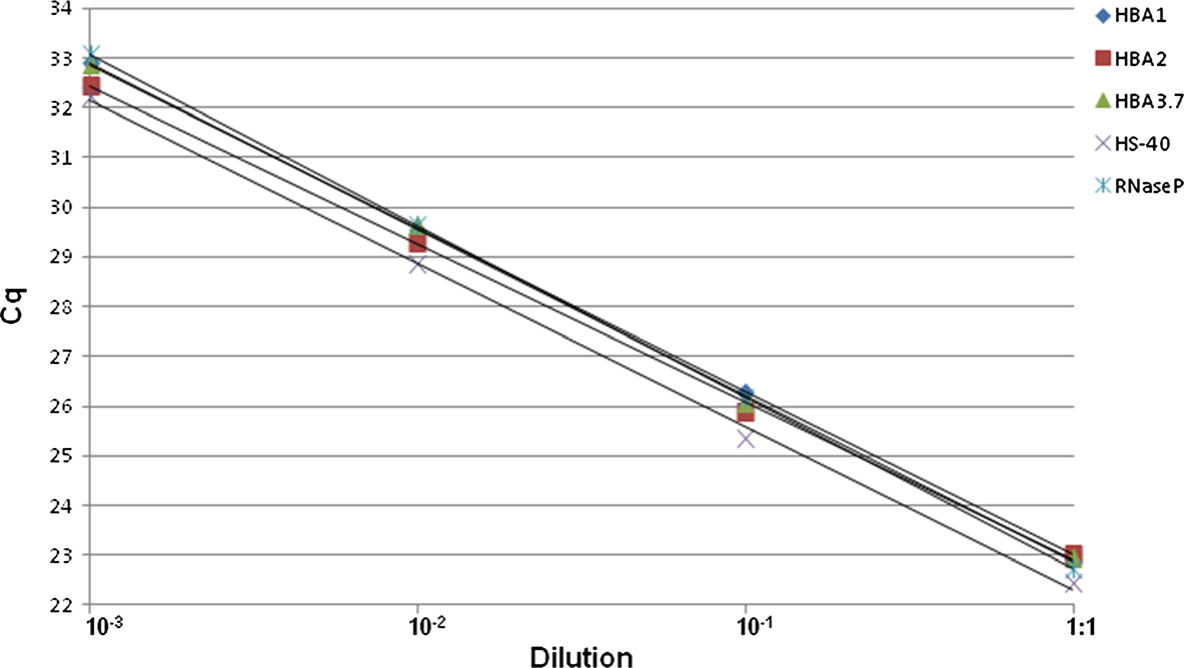
**Evaluation of efficiency of each target CNV assay and reference assay RNaseP.** Quantification cycle is plotted as a function of DNA concentration. Standard curves were generated using a 10-fold serial dilution of DNA. The resulting equations were: y = 3,2868x + 36,147, y = 3.1712x + 35.587, y = 3.3393x + 36.224, y = 3.2791x + 35.417 and y = 3.4487x + 36.523 for the HBA1, HBA2, HBA3.7, HS-40 and Rnase P assays, respectively. All curves showed a highly linear correlation (r^2^) of 1.0, 0.999, 0.999, 0.999 and 1.0, respectively.

To evaluate the efficiency of each copy number variation assay, standard curves were generated using 10-fold serially diluted DNA samples (a pool of three different samples). The slope coefficients of the standard curves of the five reactions (HBA1, HBA2, HBA3.7, HS-40 and RNaseP) were all between 3.2 and 3.4, indicating a PCR amplification efficiency of more than 97% (Figure 2). Gel electrophoresis of all PCR targets excluded the presence of primer dimers or unspecific PCR products.

A calibrator sample with two copies of both α-globin genes is necessary for correct determination of copy number in unknown samples. To achieve this, four random patient samples showing no deletions in the multiplex gap-PCR method were amplified with all four CNV assays, each sample alone and together as a DNA pool. All samples showed two copies on all four assays (results not shown). The DNA pool was used as a calibrator sample in further experiments.

**Figure 3:**
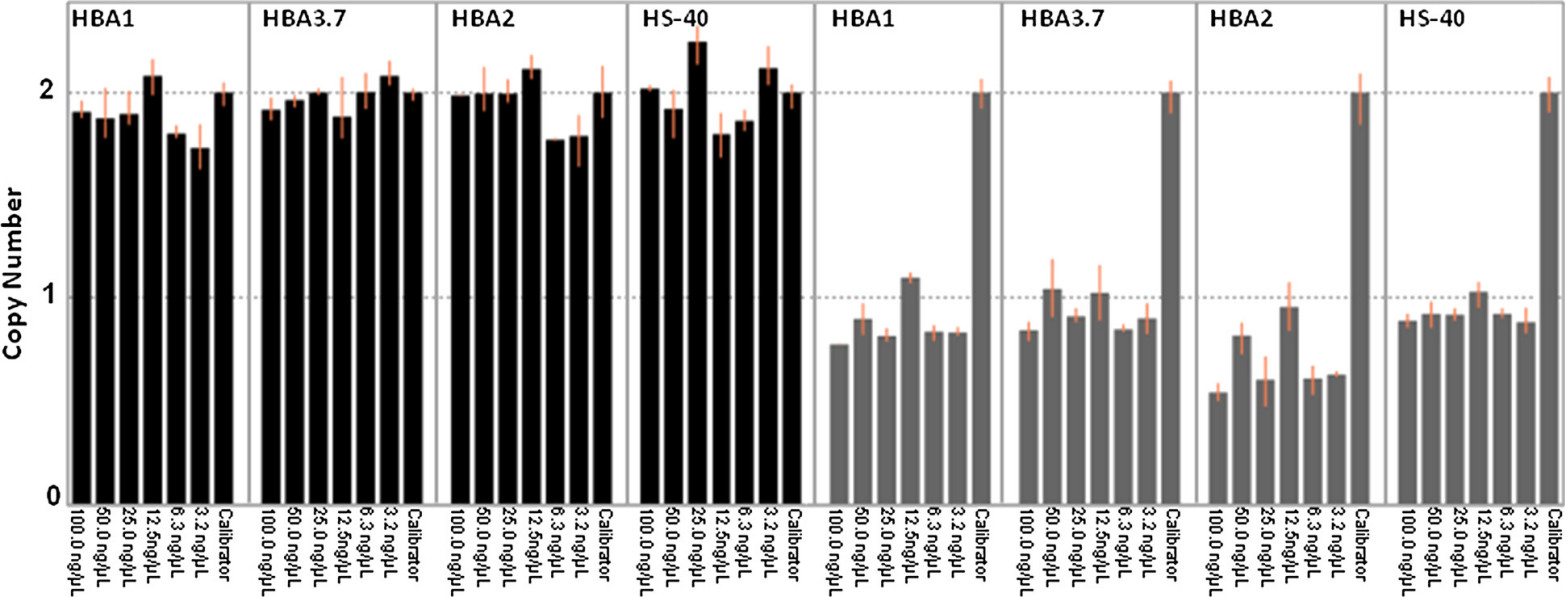
**Robustness of the copy number variation assay.** Two-fold serial dilution of a normal DNA sample (black columns) and a DNA sample with a known deletion (grey colums) were analyzed against a calibrator sample with a total concentration of 20 ng/μL. The assay reported the correct number of copies in a wide DNA concentration range from 3.2-100 ng/μL. The error bars indicates the minimum and maximum copy number calculated for each group of replicate sample.

Two-fold serial dilutions of a normal DNA sample and a DNA sample with a known deletion (αα/--^SEA^) were analyzed to determine the robustness of the HBA-CNV assay. All four assays showed the correct number of copies in a wide range of DNA template concentrations (3.2 - 100.0 ng/μL) (Figure 3).

To evaluate the newly developed CNV assays, we compared this method with a conventional gap-PCR detecting the seven most common α-thalassemia deletions [11] in 108 patient samples (Table 1). The samples comprised ten different combinations of CNVs in the α-globin region, including both normal samples and deletions of one, two and three α-globin genes. With the HBA-CNV method, we correctly identified 24 one-gene deletions, 18 two-gene deletions, including eight homozygous –α^3.7^ deletions, as well as one three-gene deletion and 63 samples without any deletion. In addition, we identified two samples with the α-globin gene triplication ααα^anti3.7^. Calculated copy numbers by the HBA-CNV analysis are shown in Table 2 for all four assays.

**Table 1:** Predicted copy number in 108 patient samples

**Genotype**	**Samples (n)**	**Copy number predicted**			
		**HBA1**	**HBA3.7**	**HBA2**	**HS-40**
αα/αα	63	2	2	2	2
-α^3.7^/αα	22	2	1	2	2
-α^4.2^/αα	2	2	2	1	2
-α^3.7^/-α^3.7^	8	2	0	2	2
--^SEA^/αα	7	1	1	1	2
--^FIL^/αα	1	1	1	1	2
-(α)^20.5^/αα	1	1	1	1	2
--^MED^/αα	1	1	1	1	2
-α^3.7^/--^SEA^	1	1	0	1	2
αα/ααα^anti3.7^	2	2	3	2	2
Total	108				

**Table 2:** Accuracy and specificity of the HBA-CNV assays

**Assay**	**Samples (n)**				**Copy number calculated (mean ± 2SD)**			
	**0 copies**	**1 copy**	**2 copies**	**3 copies**	**0 copy**	**1 copy**	**2 copies**	**3 copies**
HBA1	0	10	98	0	N/A	0,92 ± 0,22	1,86 ± 0,37	N/A
HBA3.7	9	32	65	2	0	1,10 ± 0,23	2,00 ± 0,39	2,55 ± 0,07
HBA2	0	13	95	0	N/A	0,90 ± 0,20	1,93 ± 0,47	N/A
HS-40	0	0	65	0	N/A	N/A	1,90 ± 0,27	N/A

### Detection of copy number variations in patients

**Figure 4:**
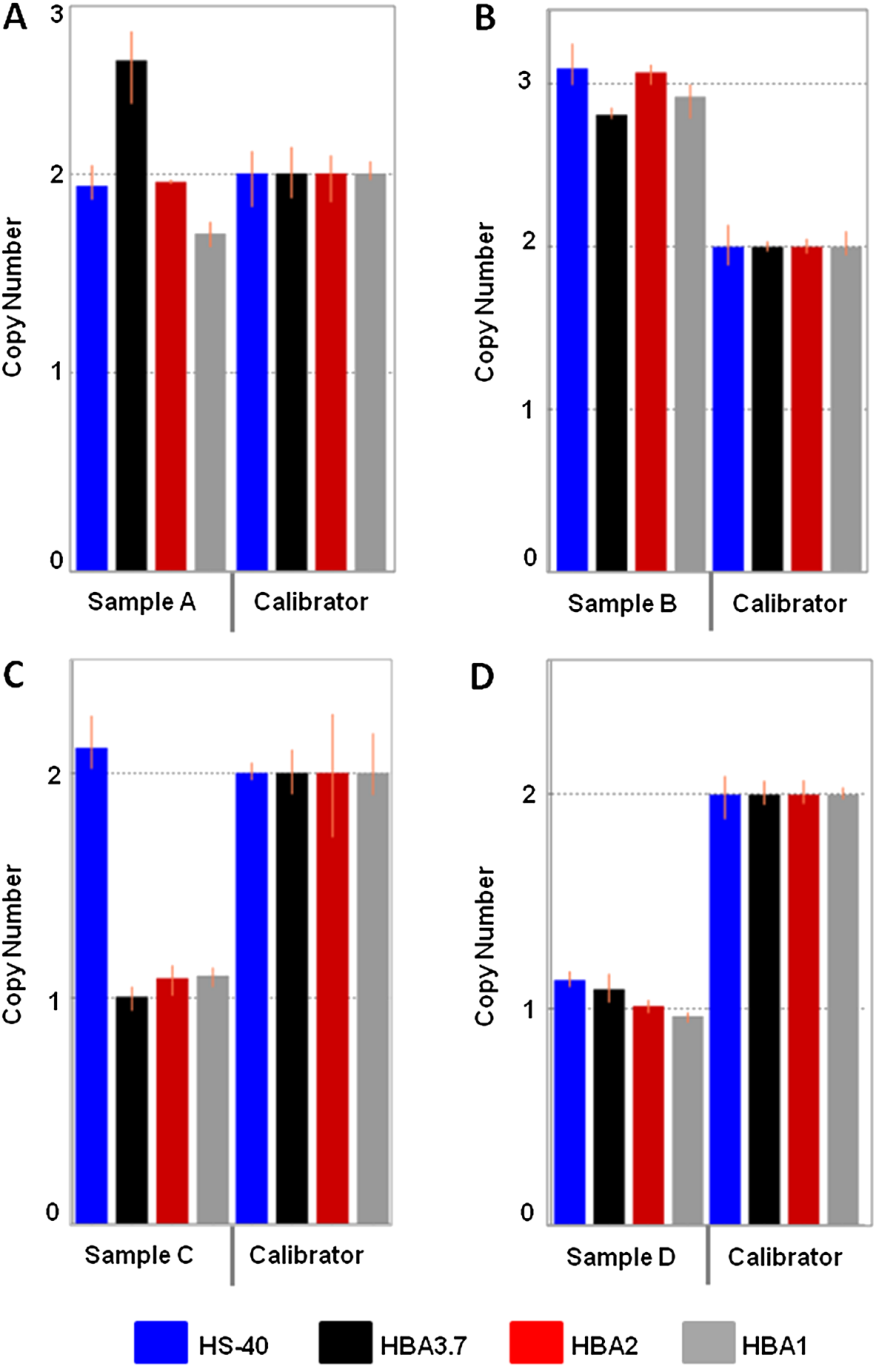
**Copy number variation in patients. A** shows data for a patient heterozygous for the α-globin gene triplication ααα^anti3.7^. **B** shows a large duplication of the complete α-globin gene cluster. **C** demonstrates a large deletion affecting both α-globin genes, and **D** shows a deletion of the HS-40 region in addition to the two α-globin genes. Control samples and non-template controls are not shown.

Since its development and validation, we have used this newly developed method in routine diagnostics of hemoglobinopathies when thalassemia is suspected from the complete blood count but not confirmed by hemoglobin HPLC or gap-PCR. Several duplications and deletions undetectable with conventional gap-PCR have been identified with the new method. As an example, we found co-inheritance of the ααα^anti3.7^-triplication in a β-thalassemia carrier with β-thalassemia intermedia phenotype (Figure 4A). Heterozygosity for a large duplication of the complete α-globin gene cluster, including the upstream regulatory element HS-40, was identified in a β-thalassemia carrier with severe hemolytic anemia (Figure 4B). The duplication was confirmed by MLPA and aCGH. The aCGH analysis showed that the duplication was approximately 170 kb in size and included the genes NPRL3, HBZ, HBM, HBA2, HBA1, LUC7 and ITFG3. Patients C and D in Figure 4 showed reduced MCV and MCH, normal HbA_2_ and HbF levels and an increased number of erythrocytes. Conventional gap-PCR was negative for both. The HBA-CNV assay profile for patient C revealed the presence of a deletion involving both α-globin genes. For patient D the HBA-CNV assay profile revealed a deletion of the complete α-globin gene cluster, including the HS-40 element.

## Discussion

Copy number variations in the form of deletions and duplications that encompass the α-globin genes can cause α-thalassemia or a more severe phenotype in β-thalassemia carriers, respectively. Traditional PCR methods are limited to detect known deletions and triplications. However, there are several cases in which patients show typical hematological changes indicating thalassemia (increased erythrocyte count, low MCV and MCH), but have normal HbA2 and HbF and negative gap-PCR. In such cases, it is important to have simple and reliable methods to identify unknown or rare deletions and duplications as part of the diagnostic armamentarium. With the implementation of the newly developed method, we were able to detect several copy number variations which had remained unidentified by traditional gap-PCR. The ααα^anti3.7^- triplications were confirmed using multiplex anti 3.7/4.2 PCR. By using MLPA and aCGH we confirmed the duplication of the complete α-globin gene cluster and estimated the size to be approximately 170 Kb. Based on the HbVar database [12], our method can be expected to detect all known larger deletions and duplications in the α-globin gene cluster and the DNase I hypersensitive site HS-40, making it superior to the traditional gap-PCR. The HBA-CNV method is, however, only able to determine the total number of copies of the target sequence, not the number on each allele. As a result, patients with ααα^anti3.7^-triplication on one allele and -α^3.7^-deletion on the other allele (ααα^anti3.7^/-α^3.7^) will be incorrectly interpreted as αα/αα, if this method is used alone. The same will happen with the ααα^anti4.2^/-α^4.2^ genotype. When the HBA-CNV method is used as a supplement to multiplex gap-PCR, this limitation will not affect the interpretation.

In our laboratory, all samples referred for hemoglobinopathy evaluation undergo standard evaluation, including complete blood count, measurement of serum or plasma ferritin concentrations, HPLC analysis of hemoglobin and multiplex gap-PCR. In cases where the hematological changes can not be explained by these standard methods, HBA-CNV and direct sequencing of the α-globin genes are performed. The HBA-CNV method and direct sequencing complement each other as sequencing detects point mutations and small deletions/insertions while HBA-CNV detects large deletions/insertions that are not revealed by sequencing. Together, HBA-CNV and direct sequencing thus provide powerful and flexible tools to identify known and novel thalassemia mutations. Our method is also useful for screening both common and unknown deletional α^0^-thalassemia. According to the guidelines of the British Committee for Standards in Haematology, an α-thalassemic status should be considered if the MCH is below 27 pg in the absence of a hemoglobin variant, β- or δβ-thalassemia heterozygosity [18]. Deletion of one α-globin gene (α^+^-thalassemia) is very common and found in many ethnic groups. Heterozygous α^+^-thalassemia is a clinically very mild form of α-thalassemia with slight microcytosis or normal hematological findings. Even if inherited in the homozygous state, α^+^-thalassemia is of no risk to a fetus [18]. The guidelines of the British Committee for Standards in Haematology therefore conclude that “α^+^ thalassemia is not regarded as significant in the screening programme and policies are designed to detect only couples at risk of hydrops fetalis” [18]. Based on this, one might argue that using only our HBA1- and HBA2 assay would detect all clinically relevant α-thalassemic deletions in the setting of a screening, making our method a rapid, reliable and cost efficient method for screening for heterozygous α^0^ thalassemia, i.e. deletions of both HBA1 and HBA2 on the same chromosome. Following this strategy, however, the consequences of overlooking an α^+^ thalassemia or a deletion of the HS-40 region need to be kept in mind. In combination with α^0^-thalassemia such mutations may lead to more severe forms of α-thalassemia, like HbH disease and hydrops fetalis, respectively.

SYBR Green real-time qPCR has been used for analysis of copy number variation in both the α- and β-globin gene clusters [13,19]. Fallah *et al.*[13] reported one of the first assays to detect unknown α-thalassemia deletions based on the relative, quantitative PCR method using SYBR Green chemistry. Five primer pairs were designed to detect CNVs affecting HBA1 and HBA2. Although this approach saves both time and cost, SYBR Green has some disadvantages that may play a crucial role when working with these genes. Because of the large degree of sequence homology between the genes in the α-globin gene cluster, assays must have high specificity and sensitivity to obtain reliable results. SYBR Green binds any double stranded DNA and this lack of specificity may cause false positive Cq values, and a false negative test result. The use of hydrolysis probes increases the specificity, and primer dimers will not influence the analysis. Compared to previously published SYBR Green methods, our approach enables a more accurate and specific copy number analysis using the gold standard real-time PCR TaqMan® probe technology. The same approach has been used in our laboratory to successfully detect deletions in the β-globin gene cluster (unpublished data). Zhou *et al.*[15] described another qPCR approach based on nested tailed-primer PCR combined with real-time qPCR. Neither of the mentioned methods investigate the erythroid-specific DNase I hypersensitive site HS-40, in which there are known mutations causing α-thalassemia [9]. By using only four target CNV assays, our method covers deletions affecting the important regulatory HS-40 region in addition to both of the α-globin genes and the common –α^3.7^ deletion. The target and reference copy number assays are run in a duplex real-time PCR reaction, and the relative quantification analysis is performed with CopyCaller™ Software using a calibrator sample with known copy number of the target sequence. Our newly developed method determines 0, 1, 2 and 3 gene copies with no overlap in calculated copy number value within two standard deviations (Table 2). Real-time qPCR involves less hands-on time and testing is simple and time-saving compared to conventional PCR and MLPA.

## Conclusion

We have developed a copy number variation analysis for detection of large deletions and duplications affecting the two α-globin genes and the regulatory region, HS-40. This method provides a flexible and simple workflow with rapid and reliable results.

## Methods

Ethylenediaminetetraacetic acid (EDTA) whole blood samples from patients referred to our laboratory for hemoglobinopathy evaluation were included. The main reasons for referral were anemia and/or microcytosis. The analysis included a complete blood count (Sysmex XE-2100, Sysmex, Japan), hemoglobin high-performance liquid chromatography (HPLC) analysis (beta-thalassemia short program, Variant, Bio-Rad, USA) and serum or plasma ferritin concentration (Cobas 8000, e602, Roche Diagnostics, Germany). We used HPLC to identify hemoglobin variants and to diagnose β-thalassemia trait when HbA_2_ ≥3.6%. Genetic testing of the α-globin genes with gap-PCR was performed in all samples with mean corpuscular volume (MCV) and/or mean corpuscular hemoglobin (MCH) below lower reference limit.

### DNA extraction

Genomic DNA was prepared from 200 μL EDTA whole blood using the MagNA Pure LC DNA Isolation Kit I according to the manufacturer’s instruction (Roche Diagnostics, Germany). Genomic DNA was eluted in a final volume of 100 μL. DNA concentrations were determined using a Nano Drop ND-1000 Spectrophotometer (Thermo Scientific, Inc. USA). The DNA was diluted to a final concentration of 50 ng/μL prior to conventional PCR and to 10 ng/μL prior to quantitative real-time PCR.

### Multiplex gap-PCR

All samples were examined for the seven most common deletions with multiplex gap-PCR using primers and conditions described elsewhere [11] with a few modifications. The PCR reaction was performed in a total volume of 25 μL using a Thermal Cycler UNO (VWR, USA). Four μL of each PCR product was analyzed by electrophoresis using 1.2% FlashGel DNA Cassettes (Lonza, Switzerland) at 200 V for 10 minutes. Pictures were taken with FlashGel Camera (Lonza).

### Multiplex anti 3.7/4.2 PCR

All samples where the new method suggested either ααα^anti3.7^ and ααα^anti4.2^ were verified with multiplex anti-3.7 and anti-4.2 PCR as described elsewhere [20] with a few modifications. The PCR reaction was performed in a total volume of 25 μL using Thermal Cycler UNO (VWR). Four μL of each PCR product was analyzed by electrophoresis using 1.2% FlashGel DNA Cassettes at 200 V for 10 minutes. Pictures were taken with FlashGel Camera.

### MLPA and aCGH

MLPA (SALSA MLPA Kit P140-B2 HBA, MCR-Holland, Netherlands) and array-Comparative Genomic Hybridization (aCGH) (SurePrint G3 Human CGH Microarray Kit, 180 k, Agilent Technologies, USA) were performed by the Department of Medical Genetics, Oslo University Hospital, Ullevaal, using standard settings.

### HBA-CNV primer and probe design

We used the HbVar track in the UCSC Genome Browser [12,21] to design four specific pairs of primers and fluorescent hydrolysis probes that would potentially detect all published large deletions in the α-globin gene cluster. To detect deletions affecting HBA1, a pre-designed TaqMan® Copy Numbers Assay, Hs03947236_cn, (Life Technologies, USA) was used. To detect the common α^3.7^-deletion and the ααα^anti3.7^ triplication, a TaqMan® copy number assay was designed to amplify a 104 bp fragment of the non-homologous region II of the α-globin gene cluster (forward primer: 5′-GGCTGTGGGCAGAGTCAGAA-3′ and reverse primer 5′-CCCCGTTGGATCTTCTCATTT-3′) together with a specific TaqMan® FAM™ dye-labeled MGB probe (5′-TGGCAGACAGGGAGG-3′). Deletions of the HBA2 gene region and the ααα^anti4.2^ triplication were identified with specific primers (5′-TCCCCTGCATCCCTTTCAG-3′ and 5′-GTAATAATCAGTGAGACTGTGGAA-3′) and a specific TaqMan® FAM™ dye-labeled MGB probe (5′-CAGTTCATTCAGCTCTG-3′) located in the non-homologous region I of the α-globin gene cluster, about 1700 bp upstream of HBA2, amplifying a 109 bp fragment. To detect copy number variations in the HS-40 region primers were designed to amplify a 101 bp fragment in intron 7 of the NPRL3 gene (specific primers 5′- GCCTCCCCCTCCTGTTTATC-3′, 5′- AGCCTGGCTGTGAACACTTTG-3′ and TaqMan® FAM™ dye-labeled MGB probe 5′- AGAGGGAAGGCCATGC-3′). RNase P was used as a reference gene and was detected by a pre-designed copy number reference assay with a VIC® dye-labeled TAMRA™ probe (TaqMan® Copy Number Reference Assay RNase P, Life Technologies, USA). RNase P is recommended as the standard reference gene in CNV experiments [22]. Custom primers and hydrolysis probes were designed using Primer Express® software, version 1.0 (Life Technologies). Pre-designed and custom assays were delivered by Life Technologies.

### Quantitative real-time PCR

Each of the HBA copy number assays were performed in a duplex real-time PCR reaction with the TaqMan® Copy Number Reference Assay for RNase P, using a Viia7 Real-Time PCR System (Life Technologies) in a standard 96 well format in a total volume of 20 μl. The PCR was carried out using 10 μl 2x TaqMan® Universal PCR Master Mix (cat. no. 4304437, Life Technologies), 1 μl 20x TaqMan® Copy Number Reference Assay RNase P (Life Technologies), 1 μl 20x TaqMan® Copy Number Assay mix, 6 μl DNase free water and 2 μl genomic DNA. The final concentration of genomic DNA was 20 ng/μL in all reactions, except for the robustness experiments. All samples were amplified in three replicates and one non-template control per primer pair was included in each run. In routine diagnostics, two control samples; a normal DNA sample and a DNA sample with a known deletion (αα/--^SEA^), are included in each run together with a calibrator sample and a non-template control. The cycling conditions comprised 10 min polymerase activation at 95°C followed by 40 cycles of 95°C for 15 s and 60°C for 1 min.

### Data analysis

The amplification curves were analyzed using Viia7 RUO software v1.2 (Life Technologies). To determine the copy number for the targets in each sample, the real-time PCR data were exported into the CopyCaller® Software (Life Technologies). The CopyCaller® software provides the calculated copy numbers and predicted copy numbers of each test sample. The software performs a comparative quantification cycle (Cq) relative quantification on the real-time data to determine the calculated copy numbers. First, the difference between the Cq of the target and reference assay is calculated (ΔCq) in each sample for both the patient sample and the calibrator sample. Then, the method compares the ΔCq values of the patient sample and the calibrator sample (ΔΔCq). With this approach, the predicted copy number of normal samples with two copies of each α-globin gene will be 2 for all assays. The predicted copy number of a sample with one gene deleted will be 1 in the respective assay, and a sample with a two-gene deletion will have a predicted copy number of 1 in all three assays, except for the HS-40 assay. Due to the mathematics of the 2^-ΔΔCq^ method, the calculated copy numbers deviates from whole numbers. Predicted copy numbers are based on the calculated copy numbers and are specified as whole numbers.

### Efficiency

Standard curves to determine the amplification efficiency were generated using 10-fold serial diluted DNA from a pool of three different samples. The known concentrations of these DNA samples were plotted on a logarithmic scale against the corresponding Cq values. The PCR efficiencies were calculated according to the slope of the standard curve and the following equation:

$$\text{Efficiency}=\left[10^{\left(-1/\text{slope}\right)}\right]-1.$$

### Ethical considerations

The study received approval from the institutional research ethics committee of Oslo University Hospital.

## Competing interests

The authors declare that they have no competing interests.

## Authors’ contributions

RMG conceived of the study, participated in the design of the study, carried out the experimental work, analyzed the data and drafted the manuscript. PU and OK participated in the discussion of the results and helped to draft the manuscript. APP participated in design of the study, discussing the results, coordination of the study and helped draft the manuscript. All authors read and approved the final manuscript.

## Pre-publication history

The pre-publication history for this paper can be accessed here:

http://www.biomedcentral.com/2052-1839/14/4/prepub
