# Correction to: Rapid and reliable detection of α-globin copy number variations by quantitative real-time PCR

**DOI:** 10.1186/s12878-019-0144-5

**Published:** 2019-10-31

**Authors:** Runa M. Grimholt, Petter Urdal, Olav Klingenberg, Armin P. Piehler

**Affiliations:** 1 Department of Medical Biochemistry, Oslo University Hospital, Ullevaal, 0424 Oslo, Norway; 2 Department of Medical Biochemistry, Oslo University Hospital, Rikshospitalet, 0424 Oslo, Norway; 3 Fürst Medical Laboratory, 1051 Oslo, Norway

**Correction to: BMC Hematol (2014) 14:4**

**https://doi.org/10.1186/2052-1839-14-4**

The copy number of the HBA1 assay for the -(α)^20.5^ deletion in the HBA-CNV method described in the original article [1] was incorrectly reported. The authors wish to note that the HBA1 assay will not be affected by the –(α)^20.5^ deletion and will show two copies (Table 1 - corrected). The 3′ breakpoint of the –(α)^20.5^ deletion is located within exon 2 of the HBA1 gene [2], leaving intact the area where the HBA1 assay is amplifying. The partial deletion of HBA1 causes a complete abolition of the gene expression, hence –(α)^20.5^ is considered as a double gene deletion. This shows that even though the HBA1 assay may show two copies, a deletion affecting both alpha-globin genes can not be excluded. Similarly, the Hb Var database contains examples of deletions that will not influence HBA2 assay copy number despite affecting both alpha-globin genes. Hence, molecular data should always be evaluated together with hematological data.

**Table 1:** Predicted copy number in 108 patient samples

Genotype	Samples (n)	Copy Number Predicted			
		HBA1	HBA3.7	HBA2	HS-40
αα/αα	63	2	2	2	2
-α^3.7^/αα	22	2	1	2	2
-α^4.2^/αα	2	2	2	1	2
-α^3.7^/-α^3.7^	8	2	0	2	2
--^SEA^/αα	7	1	1	1	2
--^FIL^/αα	1	1	1	1	2
-(α)^20.5^/αα	1	2	1	1	2
--^MED^/αα	1	1	1	1	2
-α^3.7^/--^SEA^	1	1	0	1	2
αα/ααα^anti3.7^	2	2	3	2	2
Total	108				
